# Basic Mechanisms of Arsenic Trioxide (ATO)-Induced Apoptosis in Human Leukemia (HL-60) Cells

**DOI:** 10.1186/1756-8722-3-28

**Published:** 2010-08-26

**Authors:** Clement Yedjou, Paul Tchounwou, John Jenkins, Robert McMurray

**Affiliations:** 1 Cellomics and Toxicogenomics Research Laboratory, NIH-RCMI Center for Environmental Health, College of Science, Engineering and Technology, Jackson State University, 1400 Lynch Street, Box 18540, Jackson, Mississippi, USA; 2 Department of Medicine, Division of Rheumatology and Immunology, University of Mississippi Medical Center, 2500 North State Street, Jackson, Mississippi, 39216, USA

## Abstract

**Background:**

Acute promyelocytic leukemia (APL) is a blood cancer that affects people of all ages and strikes about 1,500 patients in the United States each year. The standard treatment of APL has been based on the combined administration of all-trans retinoic acid and chemotherapy including anthracyclins and cytarabine. However, 10-20% of patients relapse, with their disease becoming resistant to conventional treatment. Recently the Food and Drug Administration has approved the use of arsenic trioxide (ATO) or Trisenox for the treatment of APL, based on clinical studies showing a complete remission, especially in relapsed patients. In a recently published study we demonstrated that ATO pharmacology as an anti-cancer drug is associated with its cytotoxic and genotoxic effects in human leukemia cells.

**Methods:**

In the present study, we further investigated the apoptotic mechanisms of ATO toxicity using the HL-60 cell line as a test model. Apoptosis was measured by flow cytometry analysis of phosphatidylserine externalization (Annexin V assay) and caspase 3 activity, and by DNA laddering assay.

**Results:**

Flow cytometry data showed a strong dose-response relationship between ATO exposure and Annexin-V positive HL-60 cells. Similarly, a statistically significant and dose-dependent increase (*p <*0.05) was recorded with regard to caspase 3 activity in HL60 cells undergoing late apoptosis. These results were confirmed by data of DNA laddering assay showing a clear evidence of nucleosomal DNA fragmentation in ATO-treated cells.

**Conclusion:**

Taken together, our research demonstrated that ATO represents an apoptosis-inducing agent and its apoptotic mechanisms involve phosphatidylserine externalization, caspase 3 activation and nucleosomal DNA fragmentation.

## Introduction

Arsenic based drugs have been used as effective chemotherapeutic agents to treat several diseases and some tumors [1]. In recent years, arsenic trioxide (ATO) has been found to have a very potent anti leukemic efficacy, especially against acute promyelocytic leukemia (APL). It has been found to produce clinical remission in a high proportion of patients with APL [2]. The Chinese first discovered that a Chinese herb was effective against APL, about 100 years ago. Workers in a university in New York City, New York, fractionated this herb, tested the fractions, and found that one fraction was active against APL. When analyzed chemically, this fraction turned out to consist of ATO [2]. The origin of this ATO is believed to be the massive pollution of the rivers in China with arsenic-laden mine tailings, that the Chinese military, who administers the mines in China, discards into the rivers while mining for valuable metals. Medical reports from China have also revealed that ATO induces clinical and hematologic responses in patients with de novo and relapsed APL [2-4]. Several studies have reported that ATO induces apoptosis in malignant cells including APL, non-Hodgkin's lymphoma, multiple myeloma, and chronic lymphocytic leukemia cells [5-7]. In addition, ATO has been found to induce apoptosis in myeloid leukemia cells such as U937 and KG-1 [8,9]. Scientific data have demonstrated that ATO induced apoptosis is associated with down-regulation of Bcl-2 gene expression, up-regulation of the expression of the proenzymes of caspase 2 and 3 and activation of both caspase 1 and 3 [5,8,9]. ATO induced apoptosis is also associated with the generation of reactive oxygen species that contribute significantly to cell killing [10-12], and inhibition of growth [13]. Previous researches have indicated that the apoptosis-inducing properties of ATO are not restricted to APL, since the viability of different cancer cell lines that originate from the same lymphoid lineage vary when exposed to various concentrations of ATO [6,14,15].

**Figure 1:**
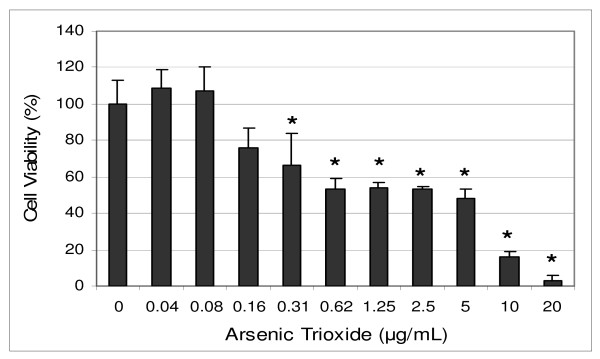
**Toxicity of arsenic trioxide to human leukemia (HL-60) cells**. HL-60 cells were cultured with different doses of arsenic trioxide for 24 hr as indicated in the Materials and Methods. Cell viability was determined based on the MTT assay. Each point represents a mean ± SD of 3 experiments with 6 replicates per dose. *Significantly different (*p <*0.05) from the control, according to the Dunnett's test [22].

Studies with APL cell lines have shown that ATO treatment activates caspases [16], down-regulates Bcl-2 protein and up-regulates of p53 expression [17]. A recent study from our laboratory has indicated that ATO induces transcription of specific genes that modulate mitogen response, cell cycle progression, programmed cell death, and cellular function in cultured HL-60 promyelocytic leukemia cells. Among these cellular responses of HL-60 cells to ATO are up-regulation of p53 tumor suppressor protein and repression of the *c-fos *transcription factor involved in cell cycle arrest or apoptosis, and modulation of *cyclin *D1 and *cyclin *A involved in cell cycle progression [18]. Preclinical studies from our laboratory have also indicated that ascorbic acid (AA), co-administrated with ATO *in vitro*, enhances ATO activity effect against human leukemia HL-60 cells [19,20], suggesting a possible future role of AA/ATO combination therapy in patients with APL. At pharmacologic doses, ATO inhibits survival and growth of several different human cancer cells in a dose- and time-dependent fashion [6,21,22]. Figure 1 shows the *in vitro *cytotoxic efficacy of ATO on human leukemia (HL-60) cells [22]. However, the specific mechanisms under which ATO exerts its therapeutic effect in cancer cells remain to be elucidated. Therefore, the aim of the present study was to elucidate the apoptotic mechanism of ATO toxicity using HL-60, a promyelocytic leukemia cell line, as a test model.

## Materials and methods

### Chemicals and test media

Arsenic trioxide (ATO), CASRN 1327-53-3, MW 197.84, with an active ingredient of 100% (w/v) arsenic in 10% nitric acid was purchased from Fisher Scientific (Houston, Texas). Growth medium RMPI 1640 containing 1 mmol/L L-glutamine was purchased from Gibco BRL products (Grand Island, NY). Fetal bovine serum (FBS), and phosphate buffered saline (PBS) were obtained from Sigma Chemical Company (St. Louis, MO). Annexin V fluorescein isothiocyanale (FITC) kit (contains annexin V FITC, binding buffer and propidium iodide [PI]), and active caspase-3 kit were obtained from BD Biosciences (Pharmingen, Becton Dickinson Co., San Diego, CA, USA).

### Cell culture

The HL-60 promyelocytic leukemia cell line was purchased from American Type Culture Collection -ATCC (Manassas, VA). This cell line has been derived from peripheral blood cells of a 36-year old Caucasian female with acute promyelocytic leukemia (APL). In the laboratory, cells were stored in the liquid nitrogen until use. They were next thawed by gentle agitation of their containers (vials) for 2 min in a water bath at 37°C. After thawing, the content of each vial of cells was transferred to a 25 cm^2 ^tissue culture flask, diluted with up to 10 mL of RPMI 1640 containing 1 mmol/L L-glutamine (GIBCO/BRL, Gaithersburg, MD) and supplemented with 10% (v/v) fetal bovine serum (FBS), 1% (w/v) penicillin/streptomycin. The 25 cm^2 ^culture flasks (2 × 10^6 ^viable cells) were observed under the microscope, followed by incubation in a humidified 5% CO_2 _incubator at 37°C. Three times a week, they were diluted under same conditions to maintain a density of 5 × 10^5 ^cells/mL, and harvested in the exponential phase of growth. The cell viability was assessed by the trypan blue exclusion test (Life Technologies Corperation, Carlsbad, CA, USA), and manually counted using a hemocytometer.

### Annexin V FITC/PI assay by flow cytometry

Annexin V FITC/PI assay for estimating early cells undergoing apoptosis was performed as described previously [20]. Briefly, 2 mL of cells (1 × 10^6^cells/mL) were added to each well of 24 plates and treated with 2, 4, 6 and 8 μg/mL of arsenic trioxide (ATO) for 24 h. Control cells were processed exactly as ATO-treated cells, except ATO treatment of these cells was eliminated. These doses were selected based on the results of previous experiments in our laboratory indicating that ATO is highly cytotoxic to HL-60 cells, showing a 24 h LD_50 _of 6.4 ± 0.7 μg/mL [22]. After 24 h of incubation, 1 × 10^6 ^cells/mL were counted and washed in PBS, re-suspended in binding buffer (10 mM Hepes/NaOH pH 7.4, 140 mM NaCl, 2.5 mM CaCl_2_), and stained with FITC-conjugated annexin V (Pharmingen, Becton Dickinson Co., San Diego, CA, USA). After staining, the cells were incubated for 15 min in the dark at room temperature. Cells were re-washed with binding buffer and analysed by flow cytometry (FACS Calibar; Becton-Dickinson) using CellQuest software [23,24].

### Active caspase-3 assay by flow cytometry

Caspase-3 assays were carried out using a commercially available kit (Phycoerythrin-Conjugated Polyclonal Active Caspase-3 Antibody Apoptosis Kits, Pharmingen). HL-60 cells were grown in RPMI 1640 containing 1 mmol/L L-glutamine (GIBCO/BRL, Gaithersburg, MD) and supplemented with 10% (v/v) fetal bovine serum (FBS), 1% (w/v) penicillin/streptomycin. Two mL of cells (1 × 10^6^cells/mL) were added to each well of 24 wells and treated with 2, 4, 6 and 8 μg/mL of arsenic trioxide (ATO) for 24 h. Control cells were processed exactly as ATO-treated cells, except ATO treatment of these cells was eliminated. Control and ATO-treated cells were assayed for caspase-3-like protease according to a previously described protocol [25]. Briefly, 1 × 10^6^cells/mL were washed per concentration with cold PBS (pH 7.4). Washed cells were suspended in Cytofix/Cytoperm solutions and incubated for 20 min on ice. Cells were pelleted and washed with Perm/Wash buffer. Cells were then centrifuged at 3000 rpm for 5 min and re-suspended in 0.2 mL Perm/Wash, 20 μL PE- conjugaled polyclonal rabbit anti-active caspase-3 antibody and incubated at room temperature for 30 min. Cells were re-suspended in 0.5 mL of perm/wash buffer and analysis by a flow cytometer (FACS Calibar; Becton-Dickinson) using CellQuest software.

### DNA fragmentation analysis by agarose gel electrophoresis

DNA fragmentation analysis was conducted to confirm the apoptotic mechanism of arsenic trioxide (ATO). Briefly, 2mL of cells (1 × 10^6^cells/mL) were added to each well of 24 wells and treated with 2, 4, 6 and 8 μg/mL of arsenic trioxide (ATO) for 24 h. Control cells were processed exactly as ATO-treated cells, except ATO treatment of these cells was eliminated. After the incubation period, cellular DNA was extracted from whole cultured cells using genomic DNA isolation reagents from Roche Molecular Biochemicals (Indianapolis, IN) according to the manufacturer's protocol. Extracted DNA samples were placed into the well of agarose gel. The agarose gels were run at 75 volts until the purple tracer marker migrated to approximately 2 cm before the end of the gel. After electrophoresis, the gel was stained with ethidium bromide, and photographed under UV light [26].

### Data analysis

Data were presented as means ± SDs. Statistical analysis was done using one way analysis of variance (ANOVA Dunnett's test) for multiple samples. Student's paired t-test was used to analyze the difference between the control and arsenic trioxide-treated cells. All p-values <0.05 were considered to be significant. Tables were constructed to illustrate the dose-response relationship with respect to annevin V and caspase-3 positive cells.

## Results

### Modulation of phosphatidylserine externalization by arsenic trioxide

**Figure 2:**
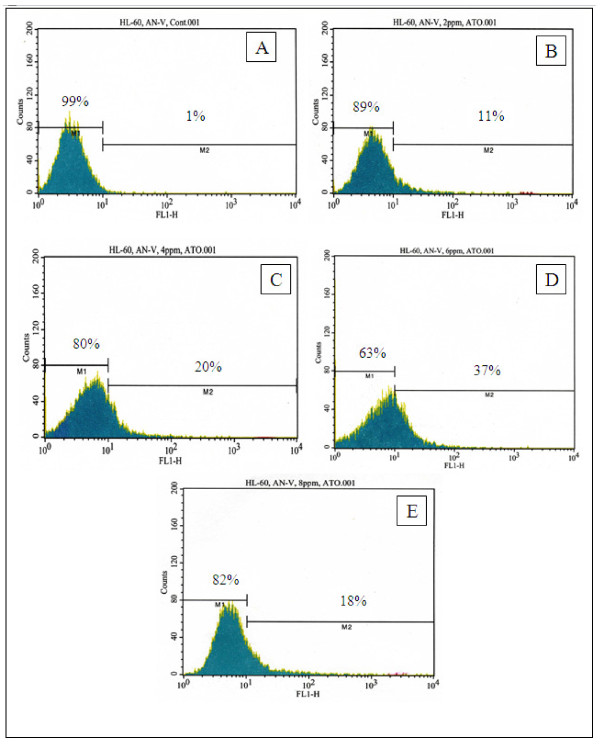
**Representative flow cytometry analysis data from Annexin V-FITC/PI assay**. The histograms show a comparison of the distribution of annexin V negative cells (M1) and annexin V positive cells (M2) after 24 h exposure to ATO. A-control; B-2 μg/mL; C-4 μg/Ml; D-6 μg/mL; E-8 μg/mL.

The response of HL-60 promyelocytic leukemia cells exposed to arsenic trioxide (ATO) was assessed by flow cytometry using Annexin V FITC/PI assay kit. As seen in Figure 2, there was a gradual increase in annexin V positive cells (apoptotic cells) in ATO-treated cells compared to the control. However, a marked and dose-dependent decrease in annexin V-positive cells was detected at 8 μg/ml of ATO, probably due to high level of cell death. The percentages of annexin V-positive cells in ATO-treated HL-60 populations were statistically significantly different compared to the percentages of annexin V cells in control group populations (Table 1). ATO-treated HL-60 cells were significantly different (*p < 0.05) *compared to the control group according to ANOVA Dunnett's test.

**Table 1:** Summary data of annexin V assay obtained from the flow cytometry analysis.

ATO Concentrations	Annexin-V Negative Cells or Viable Cells	Annexin-V Positive Cells or Apoptotic Cells
	(Mean ± SD)%	(Mean ± SD)%
0 μg/mL	99.0 ± 0.0	1.0 ± 0.0
2 μg/mL	88.5 ± .07	11.5 ± 0.7
4 μg/mL	80.4 ± 5.7*	19.6 ± 5.7*
6 μg/mL	64.2 ± 5.3*	35.8 ± 5.3*
8 μg/mL	82.4 ± 0.5*	17.6 ± 0.5*

HL-60 promyelocytic leukemia cells were cultured in the absence or presence of ATO for 24 h as indicated in the Materials and Methods. Values are shown as means ± SDs of 3 replicates per experiment. *Significantly different at *p < 0.05 *to the control group.

### Activation of caspase-3 by arsenic trioxide

**Figure 3:**
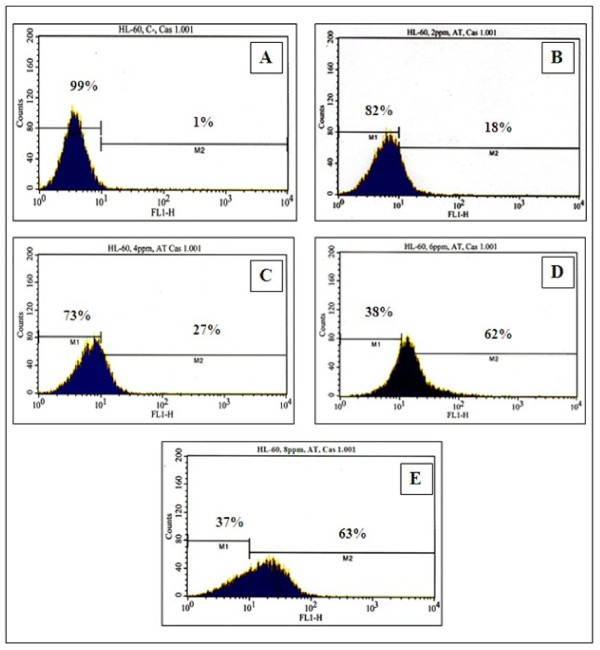
**Representative flow cytometry analysis data from active caspase-3 assay**. The histograms show the distribution of caspase-3 negative cells (M1) and caspase-3 positive cells (M2) after 24 h exposure to ATO. A-control; B-2 μg/mL; C-4 μg/Ml; D-6 μg/mL; E-8 μg/mL.

The activity of caspase-3 in HL-60 promyelocytic leukemia cells exposed to arsenic trioxide (ATO) was assessed by flow cytometry. As seen in Figure 3, there was a strong dose-response relationship between caspase-3 activation in HL-60 cells and ATO exposure. After 24 h of exposure, the percentages of caspase-3 positive cells (apoptotic cells) were 1.1 ± 0.3%, 17.5 ± 8.9%, 27.0 ± 2.4%, 62.5 ± 8.8%, and 63.1 ± 9.7% in 0, 2, 4, 6, and 8 μg/mL of ATO, respectively (Table 2). We observed significant differences (*p < 0.05*) between the control and AT-treated cells within the range of 4-8 μg/mL of ATO.

**Table 2:** Summary data of caspase-3 assay obtained from the flow cytometry analysis.

ATO Concentrations	Caspase-3 Negative Cells or Viable Cells	Caspase-3 Positive Cells or Apoptotic Cells
	(Mean ± SD)%	(Mean ± SD)%
0 μg/mL	98.7 ± 0.6	1.1 ± 0.3
2 μg/mL	82.5 ± 8.9*	17.5 ± 8.9*
4 μg/mL	63.0 ± 2.4*	27.0 ± 2.4*
6 μg/mL	37.5 ± 8.8*	62.5 ± 8.8*
8 μg/mL	36.9 ± 9.7*	63.1 ± 9.7*

HL-60 promyelocytic leukemia cells were cultured in the absence or presence of ATO for 24 h as indicated in the Materials and Methods. Values are shown as means ± SDs of 3 replicates per experiment. *Significantly different at *p < 0.05 *to the control group.

### Induction of nucleosomal DNA fragmentation by arsenic trioxide

**Figure 4:**
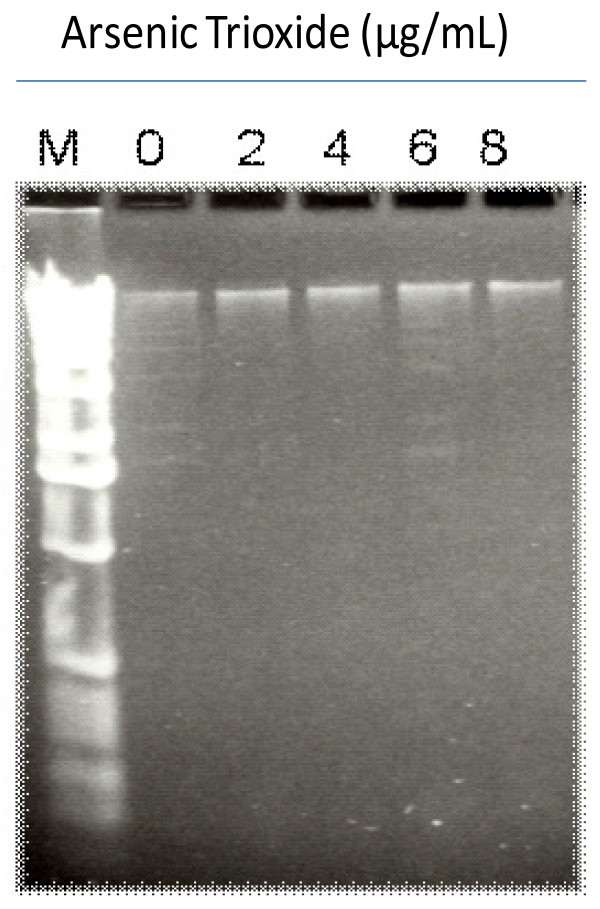
**Arsenic trioxide (ATO)-induced DNA fragmentation in HL-60 promyelocytic leukemia cells**. Lane 1: M-molecular weight marker; lane 2: control with no ATO treatment; lane 3: 2 μg/mL; lane 4: 4 μg/mL; lane 5: 6 μg/mL; and lane 6: 8 μg/mL ATO. Twelve (12) μL of each sample was electrophoresed on a 1.2% agarose. DNA was stained with ethidium bromide and then visualized under UV light.

Agarose gel electrophoresis of DNA extracted from control and arsenic trioxide (ATO)-treated cells is presented in (Figure 4). As shown on this figure, our result showed a positive nucleosomal DNA fragmentation in nuclei isolated from HL-60 promyelocytic leukemia cells. A small fragment of DNA double-strand breaks was detected in cells incubated in the absence of ATO. Overall, the present observation demonstrates that ATO exposure induced nucleosomal DNA fragmentation in HL-60 promyelocytic leukemia cells.

## Discission

Cell death is thought to take place at least by two processes that include apoptosis and necrosis. Apoptosis is an active and physiological mode of cell death. It is generally believed to be mediated by active intrinsic mechanisms, although extrinsic factors can contribute [27-30]. Apoptosis is genetically controlled and is defined by cytoplasmic and nuclear shrinkage, chromatin margination and fragmentation, and breakdown of the cell into multiple spherical bodies that retain membrane integrity [31,32]. In contrast, necrosis is an uncontrolled cell death that is characterized by progressive loss of cytoplasmic membrane integrity, rapid influx of Na^+^, Ca^2+^, and water, resulting in cytoplasmic swelling and nuclear pyknosis [33-35]. The latter feature leads to cellular fragmentation and release of lysosomal and granular contents into the surrounding extracellular space, with subsequent inflammation [30-32].

To gain insight into the mechanism of arsenic trioxide (ATO)-induced apoptosis, we examined the modulation of phosphatidylserine externalization in HL-60 promyelocytic leukemia cells. We observed that ATO induces cellular apoptosis in HL-60 promyelocytic leukemia cells in a dose-dependent manner, showing an increase expression of annexin positive cells in ATO-treated cells compared to the control. Annexin-V is a specific phosphatidylserine-binding protein used to detect apoptotic cells by providing an assessment of the progression from living cells (annexin-/PI-) towards apoptotic stage (annexin+/PI-) and postapoptotic cell death (annexin+/PI+). The effect of ATO was more pronounced at 6 μg/mL (*p < 0.05*) compared to the control cells. We observed that the percentage of annexin positive cells (apoptotic cells) increased gradually (*p < 0.05*) in a dose-dependent manner with increasing ATO concentrations and reached a maximum of (35.8 ± 5.3)% cell death after 24.h of exposure. Above 6 μg/mL exposure, ATO failed to further increase apoptosis, probably due to the high level of necrotic cell death at 8 μg/mL of exposure. From a recently published study (Figure 1), we reported that ATO is highly cytotoxic to HL-60 promyelocytic leukemia cells, showing a 24 h-LD_50 _of 6.4 ± 0.7 μg/mL [13]. Consistent with our result, previous studies have indicated that low concentrations ATO (2 μM) induces apoptosis in HPV 16 DNA-immortalized human cervical epithelial cells and its molecular pathways leading to apoptosis may be associated with down-regulation of viral oncogene expression [36].

To further gain insight into the mechanism of arsenic trioxide (ATO)-induced apoptosis, we examined caspase-3 activation in HL-60 promyelocytic leukemia cells. Caspase-3 is known as a key component of the apoptotic machinery and appears to be the most executant, which can be activated during the early and late stages of apoptosis [37]. It also a protein which has been shown to play a pivotal role in the execution phase of apoptosis induced by diverse stimuli [38]. As shown on Figure 3, we have demonstrated that ATO significantly induces apoptosis of HL-60 cells in a dose-dependent manner, at least in part, through activation of caspase-3. We have found that the percentage of caspase-3 positive cells (apoptotic cells) increases gradually with increasing ATO concentrations and reached a maximum cell death of 63.1 ± 9.7% at 8 μg/mL after 24.h of exposure. This study suggests that active caspase-3 plays an important role in executing apoptosis in ATO-treated HL-60 cells. Consistent with our results, ATO-induced apoptosis and related caspase activation have also been studied in HL-60 cells although different approaches to detect apoptosis were adopted in that study [39]. Recent studies have reported that low concentrations of ATO, in the range of clinically effective concentrations (1-5 μM), induce partial apoptosis of T lymphocytes by increasing oxidative stress and caspase activation [40]. ATO has also been shown to induce apoptosis in NB4 and mouse B cell leukemia cells [5]. One report has also indicated that arsenic-induced apoptosis in B-cell leukemia cell lines occurred through the involvement of caspases such as caspase 1 and caspase 3, and the down regulation of Bcl-2 [41]. Overall, our results indicate that active caspase-3 is involved in ATO-induced apoptosis in HL-60 cells. However, further investigations are needed to determine whether or not specific activators of caspace-3 may be directly associated with the induction of cell death.

**Figure 5:**
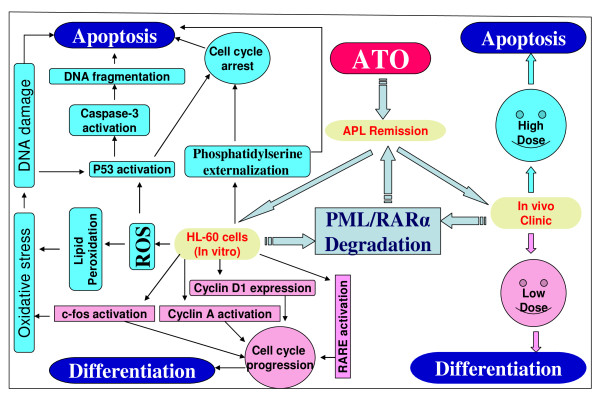
**Schematic representation of the apoptotic mechanisms of arsenic trioxide (ATO) as a therapeutic agent in the treatment of acute promyelocytic leukemia**. ATO exerts a dual effect on HL-60 cells by inducing partial differentiation and apoptosis. As shown on Figure 5, the mechanisms by which ATO induces apoptosis is mediated through oxidative stress [13] that leads to DNA damage and cell death [44], up-regulation of p53 tumor suppressor protein and repression of the *c-fos *transcription factor [18], induction of phosphatidylserine externalization, caspase-3 activation, and nucleosomal DNA fragmentation.

To confirm the apoptotic mechanism of arsenic trioxide (ATO) for the above results, we further examined the apoptotic response, as judged by the appearance of a DNA ladder through agarose gel electrophoresis. We observed DNA ladders in extracts from HL-60 cells treated with ATO at concentrations of 2, 4, 6, and 8 μg/mL for 24 h. DNA Laddering is a characteristic pattern of nucleosomal DNA fragmentation, which is the hallmark of apoptosis. DNA fragmentation is one of the later stages of apoptosis [42]. Previous researches have indicated that ATO triggers apoptosis in APL cells by degrading promyelocytic leukemia and retinoic acid receptor-a fusion protein [5,43]. *In vitro*, ATO induces apoptosis in hematological malignancies and several solid tumor cells at lower concentrations [6,15,44], and causes acute necrosis in various cell lines at higher concentrations [6]. As shown in Figure 5, a series of recently published studies in our laboratory have demonstrated that the apoptotic mechanism of ATO as an anti-cancer drug may be associated with DNA damage and cell death [45], up-regulation of p53 tumor suppressor protein and repression of the *c-fos *transcription factor [18] as result of oxidative stress [13]. A recent publication by Platanias has reported that ATO-induced cell death or apoptosis is associated with the depredation of oncoproteins, activation and suppression of pro-apoptotic and anti-apoptotic proteins respectively, generation of reactive oxygen species (ROS) which leads to the decrease in mitochondrial potential and activation of caspases in leukemia cells [46]. Together, data from annexin V assay, caspase-3 assay, and DNA fragmentation analysis collectively show that ATO induces apoptosis in HL-60 promyelocytic leukemia cells. Consistently, a recent report has indicated that ATO activates the intrinsic (mitochondrial) pathway of apoptosis, which involves the disruption of mitochondrial membrane potential, increased Bax/Bcl-2 ratio and caspase-9 activation, as well as the extrinsic death receptor pathway mediated by Fas and caspase-8 activation in acute megakaryocytic leukemia [47]. Our result is in support of previous findings indicating that ATO induces clinical remission in a high proportion of patients with APL by inducing apoptosis [2,9].

## Conclusions

We have demonstrated in the present *in vitro *study that relevant concentrations of arsenic trioxide (ATO) induce apoptosis of HL-60 promyelocytic leukemia cells. Although the exact mechanisms under which ATO exerts its therapeutic effect in APL cancer are not well elucidated, we have shown in the present study that ATO represents an apoptosis-inducing agent in HL-60 promyelocytic leukemia cells. Its apoptotic mechanisms involve the induction of phosphatidylserine externalization, caspase-3 activation, and nucleosomal DNA fragmentation.

## Competing interests

The authors declare that they have no competing interests.

## Authors' contributions

CY and PT conceived, designed and implemented the study, and drafted the manuscript.

JJ and RM participated in the implementation of the study, and the acquisition, analysis and interpretation of data. All authors read and approved the final draft of the manuscript.
